# Utility of endogenous 4β-hydroxycholesterol as a biomarker to assess cytochrome P 450 3A (CYP3A) activity: not quite ready for prime time

**DOI:** 10.1007/s00228-022-03386-z

**Published:** 2022-09-13

**Authors:** Norint P. Tung, Joseph D. Ma

**Affiliations:** Skaggs School of Pharmacy & Pharmaceutical Sciences, University of California, San Diego, 9500 Gilman Drive, La Jolla, 0657 USA

**Keywords:** 4β-hydroxycholesterol, Biomarker, CYP3A4

Kvitne et al. published an open-label, three-arm study evaluating hepatic and intestinal cytochrome P450 (CYP) 3A4 activity utilizing the 4β-hydroxycholesterol concentrations as an endogenous biomarker to evaluate CYP3A4 activity in patients with a wide body weight range (*n* = 78, BMI 18.5 to greater than 40 kg/m^2^) [1]. The authors are to be commended for evaluating organ-specific quantitative CYP3A4 protein expression and microsomal ex vivo activity. The subject participants are also to be commended for providing liver and small intestinal biopsies. 4β-hydroxycholesterol concentrations correlated with hepatic CYP3A4 concentrations (Spearman *r* = 0.3, *p* = 0.027) and with hepatic microsomal CYP3A4 activity (Spearman *r* = 0.53, *p* < 0.001). Intestinal CYP3A4 concentrations and microsomal CYP3A4 activity did not correlate with 4β-hydroxycholesterol concentrations. The authors concluded this study “…provides evidence that 4β-hydroxycholesterol concentrations is a suitable marker for hepatic CYP3A4 phenotyp[ing]” [1].

Correlation coefficients (*r*) and *r*^2^ values are commonly reported in the literature, provide evidence of an association, and are interpreted to assume suitability of a CYP phenotyping probe drug [2, 3]. However, *r* values are often overvalued, misinterpreted, provide limited information, and are not suitable in validating a phenotyping probe drug and/or endogenous biomarker for general, widespread use. The limitations of *r* and *r*^2^ values are discussed elsewhere in detail, but include the inability to measure predictive performance, whether independent (effector) variables are causes of changes in the dependent (outcome) variable, and whether omitted-variable bias exists [4–6].

Validating a phenotyping probe drug and/or endogenous biomarker requires an evaluation of predictive performance by way of assessing bias and precision. Bias represents systematic error and can be observed by over- or under-estimates of the parameter of interest (e.g., exposure or clearance). Precision is random error and represents the “effect size” of variation in a prediction [7]. Appropriate methods to determine bias and precision include visual inspection via Bland–Altman analysis or determining mean prediction error (as a measure of bias) and mean absolute error or root mean square error (as measures of precision) [4, 6]. Based on the current study, predictive performance via assessment of bias and precision needed to be evaluated between 4β-hydroxycholesterol concentrations and systemic midazolam clearance. It is interesting to note that 4β-hydroxycholesterol concentrations did not correlate with systemic midazolam clearance (Spearman *r* =  − 0.03, *p* = 0.81) [1].

We acknowledge the minimal invasiveness, ease of measurement, and the ability to discriminate the strength of CYP3A induction as advantages in using 4β-hydroxycholesterol concentrations as a biomarker. However, given the limitations of *r* and *r*^2^ values, the need to address previous concerns regarding utility [8, 9], and until proper validation steps have been performed, 4β-hydroxycholesterol concentrations are not a valid biomarker for measuring in vivo, real-time CYP3A activity. Validation criteria for CYP phenotyping probe drugs have been proposed and need to be evaluated in the content of 4β-hydroxycholesterol concentrations [2, 10, 11]. Consequently, we are concerned that the study findings may result in the inappropriate use of 4β-hydroxycholesterol concentrations in future studies evaluating CYP3A-mediated drug-drug interactions.

## Data Availability

Not applicable as no data were used.

## References

[1] Kvitne KE, Hole K, Krogstad V, Wollmann BM, Wegler C, Johnson LK, Hertel JK, Artursson P, Karlsson C, Andersson S, Andersson TB, Sandbu R, Hjelmesaeth J, Skovlund E, Christensen H, Jansson-Lofmark R, Asberg A, Molden E, Robertsen I (2022). Correlations between 4β-hydroxycholesterol and hepatic and intestinal CYP3A4: protein expression, microsomal ex vivo activity, and in vivo activity in patients with a wide body weight range. Eur J Clin Pharmacol.

[2] Fuhr U, Jetter A, Kirchheiner J (2007). Appropriate phenotyping procedures for drug metabolizing enzymes and transporters in humans and their simultaneous use in the “cocktail” approach. Clin Pharmacol Ther.

[3] Srinivas NR (2016). Prediction of area under the curve for a p-glycoprotein, a CYP3A4 and a CYP2C9 substrate using a single time point strategy: assessment using fexofenadine, itraconazole and losartan and metabolites. Drug Dev Ind Pharm.

[4] Bland JM, Altman DG (1986). Statistical methods for assessing agreement between two methods of clinical measurement. Lancet.

[5] Nagelkerke N (1991). A note on a general definition of the coefficient of determination. Biometrika.

[6] Sheiner LB, Beal SL (1981). Some suggestions for measuring predictive performance. J Pharmacokinet Biopharm.

[7] Ting LS, Villeneuve E, Ensom MH (2006). Beyond cyclosporine: a systematic review of limited sampling strategies for other immunosuppressants. Ther Drug Monit.

[8] Neuhoff S, Tucker GT (2018). Was 4beta-hydroxycholesterol ever going to be a useful marker of CYP3A4 activity?. Br J Clin Pharmacol.

[9] Penzak SR, Rojas-Fernandez C (2019). 4beta-hydroxycholesterol as an endogenous biomarker for CYP3A activity: literature review and critical evaluation. J Clin Pharmacol.

[10] Watkins PB (1994). Noninvasive tests of CYP3A enzymes. Pharmacogenetics.

[11] Zaigler M, Tantcheva-Poor I, Fuhr U (2000). Problems and perspectives of phenotyping for drug-metabolizing enzymes in man. Int J Clin Pharmacol Ther.

